# Living in LALA land? Forty years of attenuating Fc effector functions

**DOI:** 10.1111/imr.13379

**Published:** 2024-08-19

**Authors:** Geoff Hale

**Affiliations:** ^1^ mAbsolve Limited Oxford UK

**Keywords:** antibody engineering, Fc receptor, Fc region, therapeutic antibody

## Abstract

The Fc region of antibodies is vital for most of their physiological functions, many of which are engaged through binding to a range of Fc receptors. However, these same interactions are not always helpful or wanted when therapeutic antibodies are directed against self‐antigens, and can sometimes cause catastrophic adverse reactions. Over the past 40 years, there have been intensive efforts to “silence” unwanted binding to Fc‐gamma receptors, resulting in at least 45 different variants which have entered clinical trials. One of the best known is “LALA” (L234A/L235A). However, neither this, nor most of the other variants in clinical use are completely silenced, and in addition, the biophysical properties of many of them are compromised. I review the development of different variants to see what we can learn from their biological properties and use in the clinic. With the rise of powerful new uses of antibody therapy such as bispecific T‐cell engagers, antibody‐drug conjugates, and checkpoint inhibitors, it is increasingly important to optimize the Fc region as well as the antibody binding site in order to achieve the best combination of safety and efficacy.

## 1 INTRODUCTION

March 13, 2006 was a very bad day for development of therapeutic antibodies. Six healthy young men suffered severe side effects following the infusion of TGN1412, an experimental antibody designed to target the immune system for treating leukemia and autoimmune diseases. To the dismay of the investigators, the infusions caused a life‐threatening cytokine storm resulting in permanent injury to some of the volunteers.^1^, ^2^ A subsequent investigation by a group of experts, chaired by Sir Gordon Duff, produced recommendations which had a considerable impact on the subsequent conduct of first‐in‐man clinical studies.^3^

In the aftermath, scientists tried to discover what went wrong. Why did experiments in mice and monkeys not reveal the danger? TGN1412 targets the CD28 antigen on T lymphocytes, and this kind of molecule was known to induce cell proliferation and release of inflammatory cytokines. TGN1412 had been selected for its particular ability to deliver a powerful signal to T cells; it is a so‐called “super‐agonist.” Differences in the expression of CD28 between humans and cynomolgus monkeys were later put forward to explain why the dramatic adverse effects had not been seen in preclinical studies.^4^ However, cell activation also required engagement of the antibody's Fc region and the role of Fc receptors in the phenomenon seems to have been underestimated.

## 2 IMMUNOGLOBULIN SUBCLASS AND FC RECEPTORS

**FIGURE 1:**
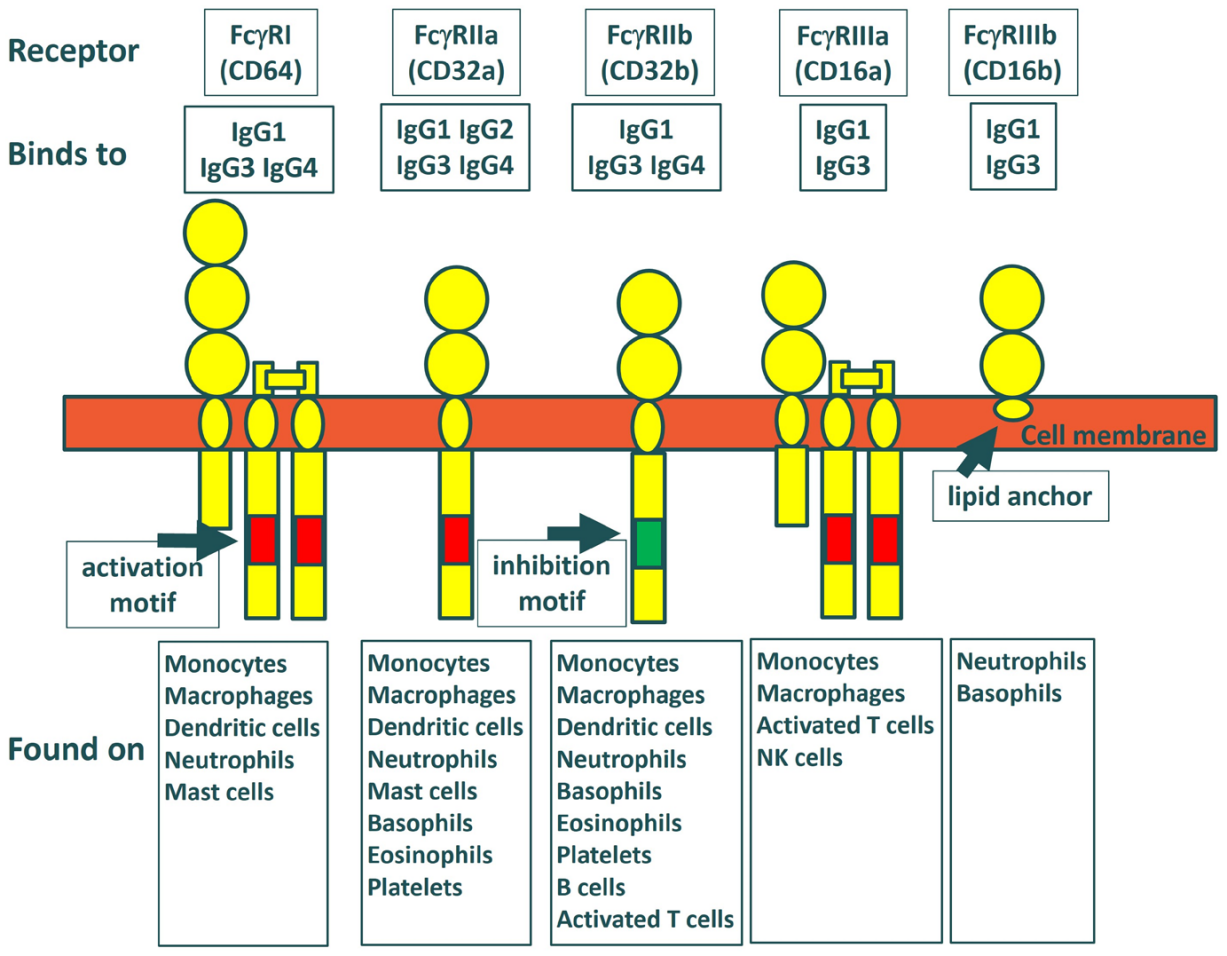
Diagram of human Fcγ receptors showing their main interactions with human IgG subclasses and expression on cells of the immune system.^7^ Alleles of FcγRIIa (131H and 131R), FcγRIIIa (158F and 158V), and FcγRIIIb (NA1 and NA1) differ in their affinity for IgGs and Fc variants.

Humans have four subclasses of IgG antibodies, of which IgG1, IgG2, and IgG4 are used in therapeutic antibodies. They interact with at least three different classes of Fcγ receptors which are found on a variety of cells of the immune system and exist in different isoforms and allotypes (Figure 1). For the purpose of this essay, I gloss over the complexity of the IgG‐FcR system. Its genetic and somatic diversity are well described in other reviews.^5^, ^6^, ^7^, ^8^ Suffice it to say that the four IgG subclasses bind with different affinities to the various Fcγ receptors, and this results in differing roles in antigen presentation, cytotoxicity, and immune regulation. As a general rule, IgG1 binds most strongly and activates all of the human Fcγ receptors, IgG2 interacts primarily with FcγRIIa while IgG4 interacts with FcγRI, FcγRIIa, and FcγRIIb. In addition, IgG1 also binds the most strongly to complement component C1q and activates complement‐dependent cytotoxicity (CDC). IgG3 also binds to Fcγ receptors and C1q and has many unique properties which I am ignoring here because it is rarely used in therapeutics.

## 3 FC SILENCING WITH IgG4

**FIGURE 2:**
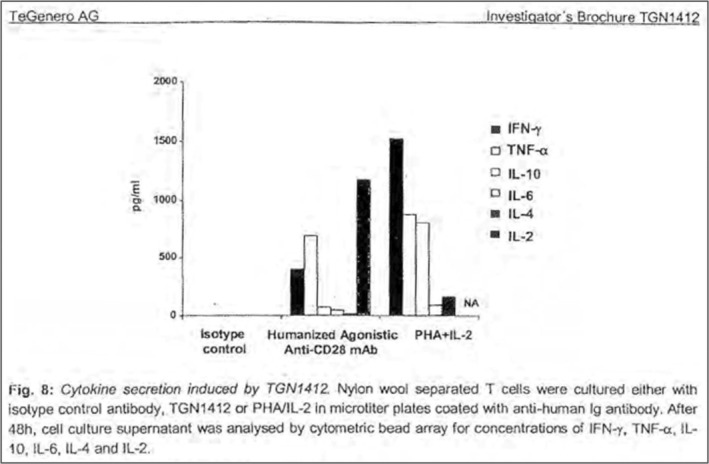
Extract from the TGN1412 Investigator's Brochure^9^ showing the high levels of INFγ, TNFα and especially IL2 which are released following incubation of T cells with immobilized TGN1412. This is one of the figures redacted from a version of the brochure that was made available to the public and the expert panel. It was only released by the MHRA following years of litigation by the Information Commissioner and others and has hitherto not been published.

TGN1412 was an IgG4 antibody. The rationale for selection of the IgG4 subclass was not explicitly stated, but was presumably because it failed to bind to FcγRIIIa and elicit cell‐mediated cytotoxicity (ADCC), in contrast to the IgG1 version, TGN1112.^9^ Human IgG4 antibodies were believed not to activate complement or mediate ADCC through FcγRIII.^10^, ^11^, ^12^, ^13^, ^14^ So, when reduction of Fc effector function was needed, the IgG4 subclass was recommended.^15^, ^16^, ^17^, ^18^ But in reality, the picture was not so simple. As early as 1993, it was shown that IgG4 could mediate ADCC in some individuals.^19^ In 1996, we compared IgG1 and IgG4 versions of alemtuzumab (Campath) in a clinical study and found that IgG4 was perfectly capable of depleting target cells and releasing inflammatory cytokines.^20^ (So far as I know, this may be the only clinical comparison of two subclasses of the same antibody.) It was unfortunate that the result was largely forgotten by the time of the catastrophic trial 10 years later. Nevertheless, the investigators did know that the Fc region of TGN1412 was necessary for T cell proliferation, since they had shown that F(ab)_2_ fragments were inactive. They also saw that TGN1412 could provoke dramatic release of the inflammatory cytokines IFN‐γ, TNFα, and IL‐2 from human blood cells (Figure 2)—though regrettably the data were suppressed for many years. It was only with substantial effort that this effect was later rediscovered by others and its predictive power was appreciated.^21^, ^22^

Our recent analysis of a matched panel of antibodies shows that IgG4 binds well to FcγRI (CD64), FcγRIIa (CD32a), and FcγRIIb (CD32b) and is very effective at inducing antibody‐dependent phagocytosis (ADCP) by any of these receptors (Table 1).^23^ But this only confirms what was already understood in the 1990s. Following the TGN1412 trial, there is little doubt that engagement of IgG4 with Fc receptors results in physiological effects that can sometimes have catastrophic consequences. It is still not clear exactly *which* receptors, though FcγRIIb on monocytes has been implicated.^24^, ^25^ This may explain the puzzling finding that a “silenced” L235E variant of TGN1412 was still active,^22^ since we shall see that the L235E mutation does not silence activity via FcγRIIb.

**TABLE 1:** Relative activity of CD20 antibodies representing wild‐type IgG1, IgG2, IgG4 and Fc variants found in approved drugs as measured by cell‐based assays for ADCP, ADCC, CDC and by DSF for thermal stability.

Isotype	Mutations	ADCP and ADCC (% of wild‐type IgG1)						CDC	DSF
		FcγRI	FcγRIIa 131H	FcγRIIa 131R	FcγRIIb	FcγRIIIa 158F	FcγRIIIa 158V	(% of IgG1)	Tm (°C)
IgG1		100.0	100.0	100.0	100.0	100.0	100.0	100.0	73.2
IgG1	E233P L234V L235A	80.6	0.6	5.2	1.9	0.9	7.3	−1.9	74.3
IgG1	L234A L235A (LALA)	78.3	27.7	29.6	35.6	2.3	27.9	−0.5	67.0
IgG1	L234A L235A G237A	−0.3	67.4	1.4	65.0	0.2	0.0	0.2	76.4
IgG1	L234A L235A P329G	0.4	0.3	0.1	−0.1	−0.2	0.1	−1.0	70.3
IgG1	L234F L235E D265A	0.0	−0.8	0.7	0.5	0.8	−0.1	20.2	67.1
IgG1	L234F L235E P331S	27.0	50.1	37.8	72.4	0.2	3.1	−1.2	66.1
IgG1	L235A G237A	3.5	76.9	7.8	75.8	0.3	0.1	14.7	73.4
IgG1	L235R G236R S239K A327G A330S P331S	22.1	1.1	17.4	0.7	−0.1	−0.2	−1.5	74.6
IgG1	N297A	81.7	−0.8	−0.9	−0.7	−0.6	−0.2	8.4	61.7
IgG1	N297G	70.3	−0.1	0.1	0.0	0.6	−0.1	−0.3	64.9
IgG2		0.3	42.0	115.3	2.9	0.1	−0.1	2.8	71.8
IgG2	A330S P331S	0.1	44.6	69.7	10.5	−0.4	−0.1	−2.7	68.7
IgG2/4	IgG2/4 chimera	1.0	16.6	15.7	0.2	0.0	0.0	2.8	71.5
IgG4		122.5	85.3	64.8	137.7	0.6	0.2	−1.3	70.3
IgG4	E233P F234V L235A G236del	−0.3	73.4	96.9	23.6	0.6	0.2	−2.2	71.0
IgG4	E233P F234V L235A D265A	−0.2	0.1	1.0	0.8	1.0	0.2	−2.3	66.8
IgG4	F234A L235A	27.9	32.2	3.8	40.6	0.4	−0.1	−1.3	69.9
IgG4	L235E	17.6	57.1	24.7	105.9	0.4	0.2	−2.4	67.8
IgG1	L234S L235T G236R (STR)	−0.3	−0.5	0.3	0.0	0.1	−0.2	−1.5	75.0

*Note*: The STR variant is included as a negative control. The activity assays are normalized and expressed as a percentage of wild‐type IgG1. Cells are shaded to indicate the response range: green = negative (<2%), yellow = significant (2%–10%), orange = substantial (10%–40%), red = high (more than 40%). For thermal stability, cells are shaded to indicate the first melting point (*T*_m_) compared with wild‐type IgG1: green = >73°C, yellow = 70°C to 73°C, orange = 66°C to 70°C, red = <66°C. Results are extracted from reference.^23^

**FIGURE 3:**
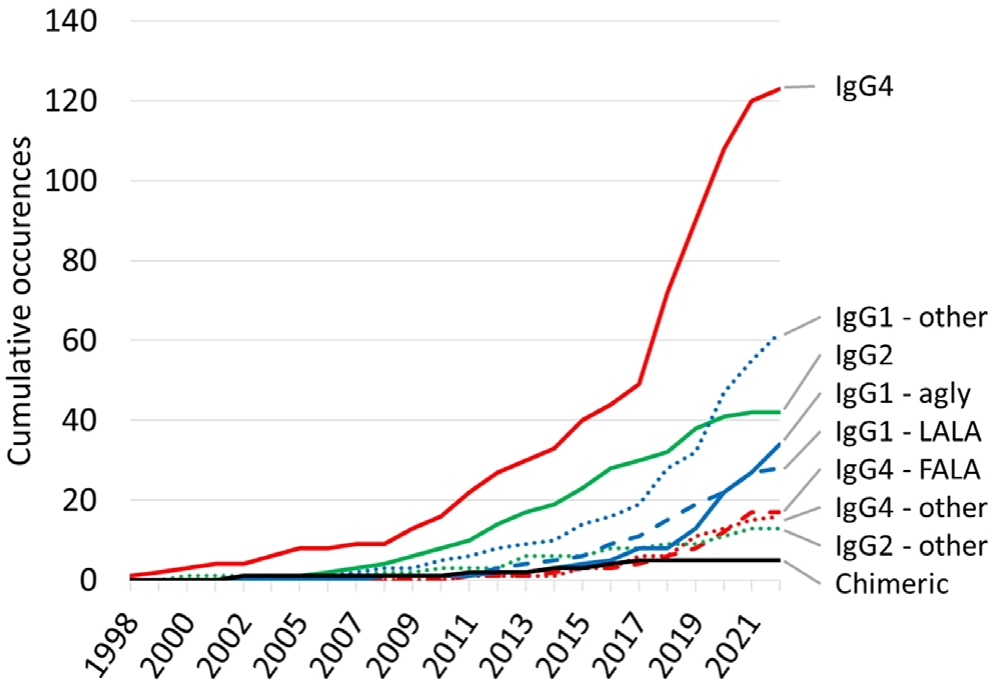
Cumulative use of different variants for Fc silencing in antibodies and Fc fusion proteins assigned an international nonproprietary name (INN). By far the most commonly used is wild‐type IgG4.^26^

In any event, we were surprised to see that IgG4 antibodies still enter the clinic in considerable numbers (Figure 3), even if they do not target such a super‐antigen as CD28.^26^ As of April 2022, 124 wild‐type IgG4 antibodies had been assigned an international nonproprietary name (INN)—which generally correlates with entering advanced clinical trials—and 21 had been granted a marketing authorisation in at least one country. (even more remarkably, 23% of these antibodies lack a stabilizing S228P mutation, though it has long been known that this is necessary to abolish Fab‐arm exchange and consequent loss of bivalency.^27^)

In 2000, gemtuzumab ozogamicin became the first IgG4 antibody to be approved for clinical use. It is an antibody‐drug conjugate (ADC) which targets the CD33 antigen on myeloid cells and is used for treatment of acute myeloid leukemia. Between 2010 and 2017, it was withdrawn from the market due to toxicities which included severe thrombocytopenia in 99% of patients.^28^ Toxicity of ADCs is a general feature of their payloads, but it seems likely that binding to FcγRIIa on platelets may be a contributory factor to the off‐target effects of this and other ADCs.^29^

Natalizumab, an antibody against α4 integrin used for treatment of multiple sclerosis, was the second IgG4 to reach the market. It was briefly withdrawn due to the occurrence of progressive multifocal leukoencephalopathy (PML) caused by reactivation of the JC virus—likely a target‐mediated effect. The drug was reinstated with suitable controls and monitoring. Otherwise, natalizumab is generally well‐tolerated without any side effects associated with binding to Fcγ receptors.

## 4 FC SILENCING WITH IgG2 AND IgG2/4 CHIMERA

Although it has received less attention, IgG2 is potentially a more “silent” subclass than IgG4. It does not bind or activate ADCP through FcγRI and shows less activity with FcγRIIa. Wild‐type IgG2 was used in 42 INNs^26^ of which 12 have been granted a marketing authorization, the first being panitumumab (anti‐EGFR) for colorectal cancer and denosumab (anti‐RANK ligand) to increase bone mass in people at high risk for fracture. The 12 licensed antibodies are used for a range of different indications, but none of them are particularly noted for severe infusion reactions which could be ascribed to unwanted FcγR binding. However, one of the complications of IgG2 antibodies is the heterogeneity caused by disulphide interchange which results in isoforms with differing activities and biological properties.^30^, ^31^ The absence of residue G236 also results in reduced binding to FcRn and a shorter half‐life.^32^

A chimeric antibody comprising the CH1 domain and hinge from Ig2 and the CH2 and CH3 domains from IgG4 is more “silent” than either alone.^33^ This construct has been used in four INNs. Two have received marketing authorisations. Eculizumab and ravulizumab both target the C5 component of complement and are used for treating diseases associated with unwanted complement activation.

## 5 AGLYCOSYL ANTIBODIES

TGN1412 was not the first antibody to a T cell receptor to induce severe cytokine release syndrome (CRS). Two decades earlier, OKT3 (muromonab), the first monoclonal antibody approved by the FDA, produced similar effects.^34^, ^35^ To mitigate the risk, OKT3 was usually administered in conjunction with high‐dose intravenous corticosteroids in an intensive care context. Even then, most recipients experienced fever, rigors, dyspnoea, vomiting, and diarrhea, and a few had life‐threatening reactions. As a result, prior to its withdrawal in 2010, OKT3 carried a “black‐box” warning in the prescribing information. Subsequent studies of OKT3 and other anti‐CD3 antibodies showed conclusively that Fc receptor cross‐linking was essential for T cell activation and cytokine release, but not for the immunosuppressive activity.^36^, ^37^, ^38^ Even the lower levels of FcγR binding of IgG2 and IgG4 versions of anti‐CD3 antibodies was sufficient to activate T cells.^39^, ^40^ Therefore, considerable effort was invested to alter the antibody Fc region to abolish Fc receptor interaction, and “Fc‐silenced” CD3 antibodies entered clinical trials.^41^, ^42^

One of the early routes to Fc silencing was the removal of the N‐linked carbohydrate which is a feature of antibody Fc regions. Some of the first clues to the role of Fc glycans had come in 1973 from studies of rabbit antibodies treated with endoglycosidase.^43^ Ten years later, Nose and Wigzell produced monoclonal antibodies deficient in carbohydrate by culturing hybridoma cells in the presence of a glycosylation inhibitor.^44^ The aglycosylated antibodies had lost the ability to activate complement, bind to Fc receptors on macrophages, or induce ADCC. Another 10 years on, and aglycosyl antibodies, including anti‐CD3, could be made by genetically engineering the heavy chain to change Asn‐297, thus removing the N‐linked glycosylation site.^40^, ^45^, ^46^ Aglycosyl anti‐CD3 was a distinct improvement compared with the wild‐type subclasses; the in vitro mitogenic effects were greatly reduced without compromise to its immunosuppressive ability and in vivo tests in mice transgenic for human CD3 showed a substantial reduction in cytokine release.^40^ Nevertheless, residual activity in a sensitive T cell assay showed that it was not completely silenced. The humanized aglycosyl anti‐CD3 was difficult to manufacture (G Hale, unpublished results), but sufficient was made for a small clinical trial to treat kidney transplant rejection.^42^ Unlike OKT3, the treatment was well tolerated with much lower levels of cytokine release and none of the severe side effects, even when high‐dose steroids were omitted. Seven of the nine patients responded well with reversal of the rejection episode.

This original aglycosyl anti‐CD3 was modified to create otelixizumab, which could be produced in greater yield, though it still suffered from aggregation at low pH (G Hale, unpublished results). Otelixizumab (also known as ChAglyCD3) was tested in a randomized trial in patents with newly diagnosed Type I diabetes.^47^ A short course of treatment (6 days) preserved beta‐cell function and significantly reduced insulin needs for more than 4 years.^48^ In this context, cytokine release was only partly suppressed. All of the patients suffered transient acute reactions which were more severe at higher doses of the drug. (Unlike the kidney transplant patients, no baseline immunosuppressive agents were being used). Partly as a consequence of these reactions, later clinical studies of otelixizumab used much lower doses, but they proved to be clinically ineffective and development was discontinued.^49^ Further confirmation that aglycosylation does not completely prevent cytokine release by CD3 antibodies came from a small study of otelixizumab in six patients with rheumatoid arthritis (CA Lawson, R Harry, B Griffiths, G Hale, H Waldmann, J Isaacs, unpublished results). All of them suffered moderate or severe reactions with high levels of TNFα and IFNγ and this was only reduced a little by dose reductions or prophylaxis with steroids. We now know that aglycosyl antibodies bind to FcγRI and mediate ADCP.^23^ Furthermore, they have greater sensitivity to proteases, reduced thermal stability and a greater tendency to aggregate compared with wild‐type IgG1,^45^, ^50^, ^51^ which likely accounts for the problems encountered in their manufacture.

Despite this early evidence that aglycosylation only partly ameliorates complications caused by unwanted FcγR binding, 36 aglycosyl antibodies had entered clinical trials by April 2022.^26^ To date, four (atezolizumab, eptinezumab, mosunetuzumab, and tarlatamab) have received regulatory approval. According to the prescribing information, infusion‐related reactions were rare for the anti‐PD‐L1 atezolizumab (1.3%) and anti‐CGRP eptinezumab (1.5%). However, mosunetzumab and tarlatamab both have “black‐box” warnings of the risk of serious or life‐threatening CRS which occurred in 39% and 55% of patients respectively. Both of these antibodies are bispecific T cell engagers with anti‐CD3 as one of the arms. As with the conventional anti‐CD3 antibodies, it appears that aglycosylation is not sufficient to eliminate unwanted inflammatory responses, even though, in the case of tarlatamab, additional mutations were introduced to restabilize the CH2 domain.^52^

## 6 FC SILENCING BY MUTATIONS IN THE LOWER HINGE REGION

In parallel with the studies on aglycosyl antibodies, investigators were finding other mutations in the Fc region which would reduce binding to Fc receptors. Between about 1984 and 1995, various lines of evidence, including cross‐species sequence comparisons, domain swapping, and site‐directed mutagenesis, led to the identification of the CH2 domain, and particularly the proximal “lower hinge” region comprising residues 233–237 as being particularly important for the binding of FcγRI and FcγRII as well as C1q.^39^, ^46^, ^53^, ^54^, ^55^, ^56^, ^57^, ^58^ The same site is also involved in the binding of FcγRIII. In those days, creation of variants by site‐directed mutagenesis was laborious and it was not yet practicable to test large panels of different or multiple amino acid alterations. Such work would later be done by teams at Genentech^59^ and Xencor.^60^ Nevertheless, effective silencing mutations were identified at residues 233, 234, 235, 236, and 237, usually by replacing the wild‐type residue with alanine or the corresponding residue from a less active subclass. The earliest, and most successful, of these single‐site mutations was IgG4 with L235E. This was a more effective way of silencing OKT3 than the wild‐type IgG4 alone.^39^ IgG4 with L235E has been used in eight INNs including sutimlimab, an anti‐C1s antibody (i.e., a complement inhibitor) which was approved in 2022 for treatment of hemolysis in cold agglutinin disease. However, infusion‐related reactions occurred in 29% of patients and sometimes required discontinuation of the drug.

## 7 SWAPPING RESIDUES BETWEEN SUBCLASSES

Mutations were then combined in various ways to produce more completely silenced variants. Among the earliest were IgG1 with E233P/L234V/L235A or IgG4 with E233P/F234V/L235A/G236del, in which residues were simply substituted with the corresponding residue (or lack of) from IgG2.^61^ Although it is not completely silenced,^23^, ^62^ the IgG1 variant is used in levilimab, an anti‐IL6 receptor antibody developed for treatment of rheumatoid arthritis which was used for the treatment of COVID‐19 with some clinical benefit and no significant adverse effects.^63^ The IgG4 variants are found in four INNs, three of which are bispecific T cell engagers, including linvoseltamab for treatment of myeloma. Unlike wild‐type IgG4, this variant shows no activity with FcγRI though it can still engage in ADCP with FcγRIIa. E233P/F234V/L235A has been combined with another silencing mutation D265A^59^ in the IgG4 antibody tislelizumab (anti‐PD‐1) which is used in the treatment of esophageal squamous cell carcinoma. This combination of mutations almost completely eliminates FcγR activities, but, as with all variants that include D265A, comes with a substantial reduction in thermal stability.^23^, ^64^

The same principle of swapping between subclasses, was used to create an IgG2 variant with substitutions from IgG4, A330S/P331S.^61^ The rationale behind these approaches was to select naturally occurring substitutions which perhaps would be less likely to provoke antidrug antibodies. Five INNs use IgG2 A330S/P331S, including one approved drug, fremanezumab (anti‐GCRP) for treatment of migraine. However, this variant barely improves on wild‐type IgG2 but is associated with reduced binding to FcRn and potentially shorter half‐life.^65^

## 8 THE LALA FAMILY

By far the most widely used of the lower hinge combination variants is IgG1 with L234A/L235A, commonly known as “LALA,” which was reported to abolish complement activity and reduce binding to FcγRI and FcγRII by 100‐fold.^66^ The first description of this combination of mutations was in an IgG4 humanized OKT3 antibody with the equivalent substitutions F234A/L235A.^67^ In this study, wild‐type IgG1 and IgG4 versions of humanized OKT3 gave T cell proliferation similar to the original mouse antibody, but the variant was more than 10,000‐fold reduced. It was well tolerated by SCID mice engrafted with human T cells but still retained the ability to prevent rejection of an allogeneic human skin graft. F234A/L235A remains the most popular IgG4 variant with 17 INNs, including six (adebrelimab, dulaglutide, galcanezumab, mirikizumab, talquetamab, teclistamab) with marketing authorisations. Adebrelimab is an anti‐PD‐1, so far approved only in China. Dulaglutide is a Fc fusion protein which targets GLP‐1 receptor for treatment of diabetes, galcanezumab is an anti‐GCRP antibody for treatment of migraine, mirikizumab is an anti‐CD23 for ulcerative colitis. Infusion‐related reactions are uncommon with these drugs. Talquetamab and teclistamab are bispecific T cell engagers for treatment of myeloma. Among other serious adverse reactions, they caused CRS in 76% and 72% of patients, respectively, and accordingly comes with black‐box warnings.

The first clinical study of an IgG1 LALA antibody, humanized OKT3 (teplizumab), was reported in 1999,^41^ just a few months before the first aglycosyl anti‐CD3. The results were very similar. Transplant rejection was reversed in five of seven patients and first‐dose reactions were minimal. For the next 20 years, teplizumab (LALA anti‐CD3) and otelixizumab (aglycosyl anti‐CD3) followed parallel journeys, both showing promise in early trials as tolerization therapy for Type I diabetes but being discontinued by their commercial sponsors due to insufficient efficacy in larger studies. But in the end, teplizumab was rescued by Provention Bio who successfully brought it to the market in 2022.^68^

LALA became the single most popular Fc silencing variant, with 28 IgG1 INNs up to April 2022. However, apart from teplizumab, only risankizumab (anti‐IL23) and spesolimab (anti‐IL36R) have to date received marketing authorizations in the USA. Both of them are used for treatment of psoriasis and neither have reported significant infusion reactions. Prolgolimab (anti‐PD‐1) is approved in Russia for treatment of melanoma, ivonescimab (bispecific anti‐PD‐1 + anti‐VEGF) is approved in China for treatment of small‐cell lung cancer, and at the time of writing, batoclimab (anti‐FcRn) was under regulatory review in China.

## 9 THE EXTENDED LALA FAMILY

Despite their popularity, we now know that LALA variants of IgG1 are incompletely silenced. They show significant binding to soluble FcγRI and the 158V allele of FcγRIIIa and high levels of ADCP and ADCC with all of the receptors apart from the 158F allele of FcγRIIIa.^23^ Furthermore, the mutated CH2 domain has reduced thermal stability compared with wild‐type IgG1. As a consequence, several investigators have added extra mutations to make the silencing more complete, and possibly to restore thermal stability.

One of the earliest approaches was to add an extra mutation in the lower hinge to create L234A/L235A/G237A.^69^ This successfully eliminates binding to FcγRI and FcγRIII and restores thermal stability but paradoxically increases cellular activation with the 131H allele of FcγRIIa and FcγRIIb. Seven INNs use this variant, including marstacimab, an antibody to tissue factor pathway inhibitor (TFPI) used for treatment of hemophilia and well tolerated,^70^ also two PD‐1 antibodies, cadonilimab and penpulimab, approved in China for cancer therapy with a third, tagitanlimab under regulatory review. Uniquely, ALX Oncology combined L234A/L235A/G237A with N297A to create the SIRPα‐Fc fusion protein evorpacept (ALX148), which targets the macrophage checkpoint inhibitor CD47.^71^ By adding mutations in the lower hinge to an aglycosyl antibody, Fc effector functions were completely eliminated, but at the cost of significantly reduced stability and potential for aggregation. There is a huge interest in antibodies and fusion proteins which target the CD47‐SIRPα interaction but clinical trials have been beset with difficulty due to the expression of CD47 on red blood cells and other normal tissues, which results in dose‐limiting toxicities. It has been suggested that a functional Fc region is required for maximal efficiency of CD47 blockade and indeed, initial studies with evorpacept alone showed minimal responses.^72^ However, elimination of Fc effector activities has allowed evorpacept to be safely used in higher doses than other CD47 inhibitors and its ability to passively block the checkpoint inhibitor potentially enhances the effect of other therapies.^73^

In 2000 and 2001, crystal structures of the complex of human IgG1 Fc with the soluble domain of FcγRIII were published.^74^, ^75^ Identification of P329 as a widely conserved and significant contact residue prompted investigations of its potential to provide more complete silencing.^76^ Single‐point mutations P329A or P329G abolished binding to C1q and reduced binding to all of the Fc receptors by up to one order of magnitude. The combination of LALA with P329G gave almost complete elimination of binding to the Fcγ receptors and no detectable ADCC. Combination of LALA with P329A is less effective.^23^ In recent years, L234A/L235A/P329G has become increasingly popular, with 10 INNs and two approved antibodies, both bispecifics. Faricimab (anti‐VEGF + anti‐Ang‐2) is used for treating age‐related macular degeneration and diabetic macular oedema by intravitreal injection, a route which would not induce systemic side effects but where inflammatory responses would be undesirable. Glofitamab (anti‐CD20 + anti‐CD3) is used for treating large B‐cell lymphoma. Although the Fc interactions are very largely silenced, it comes with a black‐box warning of the risk of CRS which was seen in 70% of patients just like other T‐cell engagers.

## 10 MORE DISTANT RELATIVES OF LALA

A CD18 antibody incorporating L235A/G237A was one of the earliest Fc silenced variants to be used in the clinic.^77^ This combination of mutations largely silences binding to FcγRI, FcγRIIIa, and the 131R allele of FcγRIIa, but retains ADCP activity with the 131H allele and with FcγRIIb. It has been used in four INNs, including vedolizumab (anti‐α4β7 integrin) used in the treatment of inflammatory bowel disease. Infusion reactions were rare (<1%) and CRS was not seen.

Substitutions other than alanine have been used at positions 234 and 235. For example, Medimmune combined the mutations L234F/L235E with the mutation P331S that had earlier been used by Armour and others.^61^, ^78^ Although the original description claims “an almost complete loss” of binding to FcγRI, FcγRIIa, and FcγRIII, the L234F/L235E/P331S variant does not perform so well in comparison with others, giving measurable levels of ADCP and ADCC with all of these receptors as well as reduced thermal stability. Nevertheless, it has been used in seven INNs, including three approved drugs (anifrolumab, durvalumab, and the combination of cilgavimab + tixagevimab). Anifrolumab (anti‐interferon receptor) is used in treatment of SLE and is generally well tolerated. Durvalumab (anti‐PD‐L1) is used in treatment of bladder cancer. Cilgavimab and tixagevimab were antibodies against SARS‐CoV‐2, and the combination was briefly approved for treatment of Covid‐19.

L234F/L235E has also been combined with D265A in three INNs, including epcoritamab, a bispecific anti‐CD20 + anti‐CD3 used for treatment of large B‐cell lymphoma. As with the other T‐cell engagers, this has a black‐box warning of the risk of CRS which occurred in 51% of patients. L234F/L235E/D265A is more completely silenced than L234F/L235E/P331S, but has a similar reduction in thermal stability compared with wild‐type IgG1.

## 11 MISCELLANEOUS OTHER VARIANTS

As described above, D265A and P331S have been combined with several other mutations. Envafolimab, a VHH‐Fc fusion protein which targets PD‐L1 and is used for treatment of cancer in China appears to be the sole example of the variant D265A/P331S. I have not found a description of its FcγR activity, but on the basis of results with the individual mutations, it seems unlikely to completely silenced and the two mutations both reduce thermal stability.

The most elaborate silencing variant used in a marketed antibody is L235R/G236R/S239K/A327G/A330S/P331S, found in crovalimab, an IgG1 antibody to complement component C5 that is used to inhibit complement in treatment of various diseases. This combination of six mutations provides almost complete silencing, but still has some residual ADCP activity with FcγRI and the 131R allele of FcγRIIa.

## 12 A NEW VARIANT WITH MORE FAVORABLE QUALITIES

**FIGURE 4:**
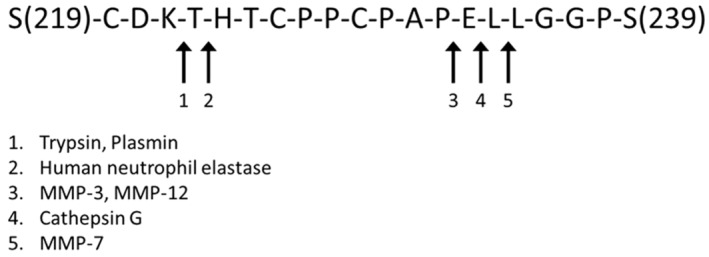
Protease cleavage sites in the hinge region of human IgG1.^79^

The lower hinge region is the most commonly targeted site for Fc silencing but many alterations compromise thermal stability (Table 1). It is also the site which is most sensitive to proteolytic degradation by matrix metalloproteases, potentially resulting in loss of biological activity (Figure 4).^79^, ^80^, ^81^ Advances in recombinant antibody technology have made it possible to prepare and screen a much larger number of variants than in the past. This allowed us to systematically explore all of the alternative substitutions at positions 234 and 235.^82^ By combining them with G236R, we discovered a set of variants which were completely silenced by all binding or functional assays—even surpassing those that were previously available, such as L234A/L235A/P327G. One of the best was L234S/L235T/G236R (“STR”). Not only was it completely silenced, its thermal stability was at least as good, if not better, than wild‐type IgG, as was its resistance to matrix metalloproteases. This variant has been adopted by the World Health Organization as the “gold‐standard” for Fc silencing. It is effective for silencing not only human IgG1, but all of the human subclasses, as well as mouse, rabbit, and monkey and also effectively silences binding to the Fc receptors of these different species, making it particularly useful for preclinical as well as clinical studies.

## 13 ANTIDRUG ANTIBODIES

It is well known that single mutations in immunoglobulin constant regions are potentially immunogenic—allotypes were discovered through the use of antisera from individuals who had been exposed to genetically different immunoglobulin.^83^ The theoretical possibility of provoking anti‐allotype responses was considered during the first administration of a humanized antibody.^84^ However, it never appears to have materialized^82^, ^85^ and even when patients had preexisting anti‐allotype antibody, the titre was not increased nor the drug neutralized.^86^ On the contrary, preexisting anti‐Fc antibodies have been reported to enhance efficacy of the therapeutic.^87^ I have not found any reports of antidrug antibodies against engineered Fc modifications, though it may be that they are not often looked for. Even an aglycosyl antibody, which likely represents a profound structural change, was apparently not immunogenic.^86^ Nevertheless, to de‐risk Fc modification, it might be appropriate to compare different mutations using in silico and in vitro methods (De Groot 2008, Jawa 2013, Brinks 2013, Harris 2024).^88^, ^89^, ^90^, ^91^ We carried out an investigation along these lines to compare a small number of variants and found that some silencing mutations have elevated scores for risk of binding to MHC Class II. Nevertheless, most of the mutations in the lower hinge region, including LALA and STR, gave no significant response in a T‐cell proliferation assay.^82^ However, many other factors contribute to immunogenicity risk, including aggregation,^90^ so it might be a good idea to consider the potential contribution of reduced stability of some variants to the overall immunogenicity risk of an antibody or fusion protein. Differential scanning fluorimetry (DSF) provides a rapid way to screen large numbers of samples and shows up the vulnerabilities of most of the existing variants.^23^

There is a possibility that elimination of binding to Fc receptors could *reduce* the overall immunogenic potential of a drug since the binding to Fc receptors on antigen‐presenting cells can lead to enhanced uptake and more effective presentation of foreign antigens.^92^, ^93^, ^94^ The underlying biology is complex and a more decisive factor may be the relative binding to activating and inhibitory receptors.^95^

## 14 WHICH VARIANT TO USE?

To date, besides wild‐type IgG2 and IgG4 antibodies, 37 antibodies and Fc fusion proteins that were genetically engineered to reduce binding and activations of Fcγ receptors have been approved or at an advanced stage of regulatory review for clinical use worldwide (Table 2). They represent 16 different variants. From an analysis of the sequences of antibodies entering clinical trials and assigned an INN, we found at least 29 other variants which have also been used in the clinic^26^ and many more have been proposed. How to choose between them? It seems that, up till now, selections have largely been based on habit (“we used that one before and it was OK”) or IP considerations (“we have a patent on this one” or “we are free to use that one”). There have been no systematic comparisons to test which are the most completely silenced and at the same time are likely to be easy to manufacture and characterize, stable, and show favorable pharmacokinetics. We have now started to address these questions by synthesizing a matched set of antibodies, all with the identical anti‐CD20 binding region, but with the whole range of Fc variations.^23^ A summary of key results for wild‐type antibodies and 16 variants found in approved drugs, together with the negative control STR, is shown in Table 1.

**TABLE 2:** List of antibody drugs approved or under review which have Fc modifications to reduce effector activity.

INN	Country of first approval	Year of first approval	Indication first approved or reviewed	Format	Target A	Target B	Isotype	Silencing mutations
Adebrelimab	China	2023	Small cell lung cancer	IgG	PD‐L1		IgG4	F234A L235A
Anifrolumab	USA	2021	Systemic lupus erythematosus	IgG	IFNAR1		IgG1	L234F L235E P331S
Atezolizumab	USA	2016	Bladder cancer	IgG	PD‐L1		IgG1	N297A
Batoclimab	China	review	Masthenia gravis	IgG	FcRn		IgG1	L234A L235A
Cadonilimab	China	2022	Cervical cancer	IgG‐scFv	PD‐1	CTLA4	IgG1	L234A L235A G237A
Cilgavimab	EU	2022	COVID‐19	IgG	SARS‐CoV‐2		IgG1	L234F L235E P331S
Crovalimab	China	2024	Atypical hemolytic uremic syndrome	IgG	C5		IgG1	L235R G236R S239K A327G A330S P331S
Dulaglutide	USA	2014	Type 2 diabetes	Fc fusion protein	GLP‐1R		IgG4	F234A L235A
Durvalumab	USA	2017	Bladder cancer	IgG	PD‐L1		IgG1	L234F L235E P331S
Eculizumab	USA	2007	Paroxysmal nocturnal hemoglobinuria	IgG	C5		IgG2/4	IgG2/4 chimera
Envafolimab	China	2021	Advanced solid tumors	dAb‐Fc	PD‐L1		IgG1	D265A P331G
Epcoritamab	USA	2023	Diffuse large B‐cell lymphoma	Bispecific IgG	CD20	CD3	IgG1	L234F L235E D265A
Eptinezumab	USA	2020	Migraine	IgG	CGRP		IgG1	N297A
Faricimab	USA	2022	Macular degeneration	Bispecific IgG	VEGF	Ang‐2	IgG1	L234A L235A P329G
Fremanezumab	USA	2018	Migraine	IgG	CGRP		IgG2	A330S P331S
Galcanezumab	USA	2018	Migraine	IgG	CGRP		IgG4	F234A L235A
Glofitamab	Canada	2023	Diffuse large B‐cell lymphoma	Fab‐IgG	CD20	CD3	IgG1	L234A L235A P329G
Ivonescimab	China	Review	Non‐small cell lung cancer	IgG‐scFv	PD‐1	VEGF	IgG1	L234A L235A
Levilimab	Russia	2020	COVID‐19	IgG	IL‐6R		IgG1	E233P L234V L235A
Linvoseltamab	USA	Review	Multiple myeloma	Bispecific IgG	BCMA	CD3	IgG4	E233P F234V L235A G236del
Marstacimab	USA	2024	Hemophilia	IgG	TFPI		IgG1	L234A L235A G237A
Mirikizumab	Japan	2023	Ulcerative colitis	IgG	IL‐23		IgG4	F234A L235A
Mosunetuzumab	EU	2022	Follicular lymphoma	Bispecific IgG	CD20	CD3	IgG1	N297G
Penpulimab	China	2021	Metastatic nasopharyngeal carcinoma	IgG	PD‐1		IgG1	L234A L235A G237A
Prolgolimab	Russia	2020	Melanoma	IgG	PD‐1		IgG1	L234A L235A
Ravulizumab	USA	2018	Paroxysmal nocturnal hemoglobinuria	IgG	C5		IgG2/4	IgG2/4 chimera
Risankizumab	Japan	2019	Plaque psoriasis	IgG	IL‐23		IgG1	L234A L235A
Spesolimab	USA	2022	Generalized pustular psoriasis	IgG	IL‐36R		IgG1	L234A L235A
Sutimlimab	USA	2022	Cold agglutinin disease	IgG	C1s		IgG4	L235E
Tagitanlimab	China	Review	Nasopharyngeal carcinoma	IgG	PD‐L1		IgG1	L234A L235A G237A
Talquetamab	USA	2023	Multiple myeloma	Bispecific IgG	GPCR5D	CD3	IgG4	F234A L235A
Tarlatamab	USA	2024	Small cell lung cancer	Tandem scFv‐scFv	DLL3	CD3	IgG1	N297G
Teclistamab	EU	2022	Multiple myeloma	Bispecific IgG	BCMA	CD3	IgG4	F234A L235A
Teplizumab	USA	2022	Type 1 diabetes	IgG	CD3		IgG1	L234A L235A
Tislelizumab	China	2019	Esophageal squamous cell carcinoma	IgG	PD‐1		IgG4	E233P F234V L235A D265A
Tixagevimab	EU	2022	COVID‐19	IgG	SARS‐CoV‐2		IgG1	L234F L235E P331S
Vedolizumab	USA	2014	Ulcerative colitis, Crohn disease	IgG	Integrin α4β7		IgG1	L235A G237A

*Note*: Data were compiled from reference.^26^, ^117^

It turns out that the familiar “LALA” variant is still active in ADCP and ADCC with all of the human Fcγ receptors. Most of the other variants, as well as wild‐type IgG2 and IgG4, have ADCP activity with FcγRI and/or FcγRII receptors similar to wild‐type IgG1. Only two variants were completely silenced in all of the function assays: IgG1 L234A/L235A/P329G and IgG4 E233P/F234V/L235A/D265A. Unfortunately, both of them show a significant reduction in thermal stability compared with wild‐type IgG1, as do most of the other variants, particularly N297A and N297G which lack the Fc carbohydrate.

The degree to which silencing is necessary depends on a host of factors: the clinical indication, the antibody format, target antigen, dose, route of administration, and so on. Antibodies which target T cell co‐receptors such as CD3 and CD28 are at the highest risk of provoking cytokine release and severe infusion reactions and these may not be completely ameliorated even with total Fc silencing. On the other hand, for antibodies which are intended simply to neutralize soluble factors, a low level of interaction may be tolerable, even though they do not need to engage with Fc receptors.

A comprehensive review in of the adverse effects associated with 110 approved antibodies found that infusion reactions had been recorded for almost 50% of them.^96^ Usually, they were mild to moderate or controllable by premedication. Reactions were more common for cancer indications, with eight antibodies having an FDA black‐box warning and 22 a warnings and precautions notice. The corresponding numbers for non‐cancer indications were one and nine, respectively. A similar analysis of 123 human or humanized IgG antibodies approved in the USA or Europe up till July 2024 is shown in Table 3. Warnings about cytokine release or infusion reactions were significantly more frequent (*p* < 10^−6^) for antibodies against cell‐surface antigens (77%) than for antibodies against soluble antigens (29%). Remarkably, among the antibodies against cell‐surface antigens, warnings of CRS and total warnings were significantly *more* frequent (*p* = 0.01, *p* = 0.04 respectively) among the “silenced” antibodies compared with the “wild‐type” antibodies. There were six “silenced” antibodies with warnings of CRS, all of which target CD3 either alone (teplizumab) or as a component of a bispecific antibody (epcoritamab, glofitamab, mosunetuzumab, talquetamab, teclistamab). Teplizumab, which uses the original LALA mutations, gives CRS in only 5% of patients, whereas the bispecific antibodies, which generally use more effective silencing mutations, have a frequency between 39% and 76%. It seems likely that Fc silencing might never eliminate the risk of CRS with bispecific T‐cell engagers, because the CD3 receptor can still be cross‐linked as a consequence of engagement with the tumor cell through the other arm of the antibody. Nevertheless, there are other benefits from Fc silencing of bispecific T‐cell engagers. In mouse models, antibodies with an intact Fc region became sequestered in the lungs or depleted in the circulation and failed to achieve antitumor effects, in contrast to a Fc‐silenced antibody Wang et al.^97^

**TABLE 3:** Analysis of the frequency of warnings about cytokine release syndrome (CRS) or infusion reactions for FDA‐approved therapeutic antibodies.

Target	Isotype	Cytokine release syndrome (CRS)			
		Wild‐type		Silenced	
		No warning	Warning	No warning	Warning
Cellular	IgG1	33	13	4	5
	IgG2	6	1	0	0
	IgG4	11	1	1	2
Soluble	IgG1	20	1	4	0
	IgG2	4	0	1	0
	IgG2/4	2	0	0	0
	IgG4	7	0	2	0
Microbial	IgG1	5	0	0	0
Total		88	16	12	7

Target	Isotype	CRS and/or infusion reactions			
		Wild‐type		Silenced	
		No warning	Warning	No warning	Warning
Cellular	IgG1	12	34	0	9
	IgG2	3	4	0	0
	IgG4	3	9	0	3
Soluble	IgG1	16	5	2	2
	IgG2	4	0	1	0
	IgG2/4	0	2	0	0
	IgG4	5	2	1	1
Microbial	IgG1	3	2	0	0
Total		46	58	4	15

*Note*: Data were compiled from the Antibody Society list of approved antibodies^117^ and summaries of prescribing information. Comparisons between observed and expected values were made using the chi‐squared test, *p* values are reported in the text.

## 15 WHAT CAN WE LEARN FROM ANTIBODIES TO PD‐1 AND PD‐L1?

Experiments suggest that engagement with Fcγ receptors compromises the antitumor activity of PD‐1 antibodies but augments the antitumor activity of antibodies to its ligand PD‐L1.^98^ Zhang and coworkers directly compared a PD‐1 antibody having wild‐type IgG4 Fc (which binds FcγRI and FcγRII) with one having E233P/F234V/L235A/D265A mutations (to completely silence binding).^99^ The silenced antibody showed blocking activity in vitro, whereas the wild‐type antibody was activating. The silenced antibody also showed significant growth inhibition in mice transplanted with human tumor cells, whereas the wild‐type antibody did not. This suggested that silencing was critical for efficacy and they advanced tislelizumab to the clinic to be approved in China in 2019 and in the USA in 2024. A further argument for complete silencing of PD‐1 antibodies comes from the suggestion that the paradoxical response of “hyperprogression” in some patients might be related to cytotoxicity mediated by antibody binding to the high affinity 158V allele of FcγRIIIa.^100^, ^101^

The Antibody Society lists 18 PD‐1 antibodies that are approved for clinical use or under regulatory review (Table 4). They include four IgG1 with “silencing” mutations (LALA or LALA plus G237A), 13 wild‐type IgG4 (with S228P for stabilization), and tislelizumab. Could this set provide data to help us understand the impact of different subclasses and variants in a comparatively homogenous clinical context? The clinical picture is not yet clear. Sun and colleagues carried out a meta‐analysis of 19 clinical studies of six of them.^102^ Five were wild‐type IgG4 (camrelizumab, nivolumab, pembrolizumab, sintilimab, and zimberelimab) and only tislelizumab was Fc silenced. It was not significantly different from the wild‐type IgG4 antibodies in either adverse effects or efficacy, though some studies suggest that hyperprogression might be less common.^103^ This will be an area to watch as more data become available. For example, penpulimab is a Fc‐silenced IgG1 which is said to have a more favorable safety profile than previous PD‐1 antibodies.^104^

**TABLE 4:** List of antibody drugs approved or under review which target PD‐1 or PD‐L1.

INN	Country of first approval	Year of first approval	Format	Target A	Target B	Isotype	Silencing mutations
Cadonilimab	China	2022	Bispecific IgG	PD‐1	CTLA4	IgG1	L234A L235A G237A
Camrelizumab	China	2019	IgG	PD‐1		IgG4	
Cemiplimab	USA	2018	IgG	PD‐1		IgG4	
Dostarlimab	EU	2021	IgG	PD‐1		IgG4	
Enlonstobart	China	Review	IgG	PD‐1		IgG4	
Iparomlimab	China	Review	IgG	PD‐1		IgG4	
Ivonescimab	China	2024	Bispecific IgG	PD‐1	VEGF	IgG1	L234A L235A
Nivolumab	USA	2014	IgG	PD‐1		IgG4	
Pembrolizumab	USA	2014	IgG	PD‐1		IgG4	
Penpulimab	China	2021	IgG	PD‐1		IgG1	L234A L235A G237A
Prolgolimab	Russia	2020	IgG	PD‐1		IgG1	L234A L235A
Pucotenlimab	China	2022	IgG	PD‐1		IgG4	
Retifanlimab	USA	2023	IgG	PD‐1		IgG4	
Serplulimab	China	2022	IgG	PD‐1		IgG4	
Sintilimab	China	2018	IgG	PD‐1		IgG4	
Tislelizumab	China	2019	IgG	PD‐1		IgG4	E233P F234V L235A D265A
Toripalimab	China	2018	IgG	PD‐1		IgG4	
Zimberelimab	China	2021	IgG	PD‐1		IgG4	
Adebrelimab	China	2023	IgG	PD‐L1		IgG4	F234A L235A
Atezolizumab	USA	2016	IgG	PD‐L1		IgG1	N297A
Avelumab	USA	2017	IgG	PD‐L1		IgG1	
Benmelstobart	China	Review	IgG	PD‐L1		IgG1	
Cosibelimab	USA	Review	IgG	PD‐L1		IgG1	
Durvalumab	USA	2017	IgG	PD‐L1		IgG1	L234F L235E P331S
Envafolimab	China	2021	VHH‐Fc	PD‐L1		IgG1	D265A P331S
Socazolimab	China	2023	IgG	PD‐L1		IgG1	
Sugemalimab	China	2021	IgG	PD‐L1		IgG4	
Tagitanlimab	China	Review	IgG	PD‐L1		IgG1	L234A L235A G237A

Note: Data were compiled from reference.^26^, ^117^

On the other side of this checkpoint axis, the Antibody Society lists 10 PD‐L1 antibodies that are approved or under review (Table 4). They include four wild‐type IgG1, one wild‐type IgG4, and five different Fc variants. Of the three which have to date received FDA approval, the prescribing information records a substantially higher incidence of infusion reactions (25%) for avelumab (wild‐type IgG1) compared with the “silenced” antibodies atezolizumab (1.3%) or durvalumab (2.2%). With a spectrum of different subclasses and variants entering the clinic, it should become possible to test whether clinical activity is augmented by binding of PD‐L1 antibodies to Fcγ receptors as predicted by the experiments in mice.

## 16 OTHER IMMUNOSTIMULATORY ANTIBODIES

A recent review^101^ provides an overview of the role of Fc interactions for a range of immune checkpoints and their ligands. Besides PD‐1 and PD‐L1, it covers CD47, CD40, and 4‐1BB among others. There is a trend to select totally Fc silent antagonists for inhibitory receptors like PD‐1 or LAG‐3. But the situation is more complex for antigens expressed on tumor cells such as PD‐L1 or CD47 or activating receptors such as 4‐1BB. The field is still open!

## 17 ANTIBODY‐DRUG CONJUGATES

After a rather shaky start, ADCs have come to prominence for cancer therapy in recent years. The Antibody Society lists 12 ADCs which are FDA‐approved, plus seven others under review or approved elsewhere. There are 17 IgG1 and two IgG4. None of them are Fc silenced—in fact one (belantamab mafodotin) has enhanced Fc activity as a consequence of removal of fucose from the Fc carbohydrate. And among 70 ADCs which have been given an INN, there are 64 IgG1, two IgG2, three IgG4, and just one drug (mirzotamab clezutoclax) with the LALA mutations (I Wilkinson, personal communication). Attachment of a cytotoxic drug was seen as a way to improve on the cytolytic activity of the naked antibody, so it was logical to retain or even improve upon the native effector functions. However, this might not be the best strategy. It is estimated that only about 0.1% of an ADC is typically taken up by the tumor^28^—what happens to the rest? Severe side effects, including lymphopenia, neutropenia, thrombocytopenia, and neuropathies are common to all ADCs.^105^ At least some of these off‐target effects could well be caused by cellular uptake mediated by Fcγ and/or FcRn receptors. For example, thrombocytopenia associated with trastuzumab emtansine is reported to be a consequence of binding to FcγRIIa on megakaryocytes.^106^ The responsibility of Fc receptors was disputed^107^ but other studies suggest that it is the uptake of ADC aggregates by FcγR‐positive cells which is responsible for off‐target toxicity.^29^, ^108^, ^109^ This might be alleviated with recent drug conjugation technology to reduce the risk of aggregation, but Fc silencing would provide a more reliable solution. In any event, it seems that the potential of Fc silencing to reduce the unwanted toxicity of ADCs is a subject which is seriously underexplored^110^ even if it is unlikely to solve all of the toxicological issues.^111^

## 18 PERSPECTIVE

La La Land was a place of dreams, of what might have been.^112^ Our LALA landscape is likewise, complex, and ever shifting. A vast number of different Fc variants have been proposed, and as we have seen, many have been brought into the clinic. But there have been few systematic comparisons of their properties and fitness for purpose. The diversity and polymorphism of Fcγ receptors makes it difficult to know which variants are the most relevant for the therapeutic or adverse effects. For example, high‐affinity FcγRI is likely to be saturated by endogenous IgG. Does this mean that it is irrelevant to therapeutic antibodies present in comparatively tiny amounts? The affinity of Fc‐FcR interactions is greatly increased by aggregation. This can occur inadvertently, as a consequence of the structure or formulation of a drug, or as a result of binding to a multivalent target, be it a pathogen or host cell. In some situations, such as for the neutralization of cytokines or other soluble mediators, the degree of Fc silencing may be less critical. In others, certainly for antibodies to T cell receptors such as CD3 and CD28, perhaps for ADCs and bispecific T cell engagers, it may be very important.

But too often the choice has not been scientifically informed. Intellectual property considerations have no doubt been a factor. The patent landscape of Fc modifications is notoriously complicated. Patents up till 2019 pertaining to IgG4 antibodies have been summarized^113^ but a similar analysis is not available for other subclasses. Some of the most effective silencing mutations in the LALA family (e.g., L234A/L235A/P329G, L234F/L235E/D265A) are proprietary and use appears to be limited to the owners. Patent applications for other variants continue to be prosecuted. This situation may have encouraged some to explore more exotic regions of the Fc landscape.

With the STR variant, we have probably reached the limit of Fcγ silencing that is both complete and without compromise to the structural integrity of the Fc region.^82^ Nevertheless, there is still plenty of scope for antibody engineers to exercise their ingenuity. Important advances are being made in the modulation of FcRn binding to control the pharmacokinetics of antibodies for a diverse range of therapeutic applications.^114^, ^115^ There are also numerous modifications which enhance the physiological activity of antibodies, both cellular and humoral, but many of the current ones also destabilize the structure.^116^ Hopefully, there are still many good days ahead for further developments in antibody therapy.

## CONFLICT OF INTEREST STATEMENT

Geoff Hale is a director and shareholder in mAbsolve Limited which owns and licenses the STR silencing technology.

## Data Availability

The data that support the findings of this study are available at https://doi.org/10.1080/19420862.2022.2123299 and https://www.antibodysociety.org/resources/approved‐antibodies/.
